# Rates of Sudden Unexpected Infant Death Before and During the COVID-19 Pandemic

**DOI:** 10.1001/jamanetworkopen.2024.35722

**Published:** 2024-09-26

**Authors:** Emma G. Guare, Rong Zhao, Paddy Ssentongo, Erich K. Batra, Vernon M. Chinchilli, Catharine I. Paules

**Affiliations:** 1 Penn State College of Medicine, Hershey, Pennsylvania; 2 Division of Biostatistics and Bioinformatics, Department of Public Health Sciences, Penn State College of Medicine, Hershey, Pennsylvania; 3 Department of Public Health Sciences, Penn State College of Medicine and Milton S. Hershey Medical Center, Hershey, Pennsylvania; 4 Department of Medicine, Penn State Health Milton S. Hershey Medical Center, Hershey, Pennsylvania; 5 Division of Infectious Diseases, Department of Medicine, Penn State Health Milton S. Hershey Medical Center, Hershey, Pennsylvania; 6 Department of Pediatrics, Penn State Health Milton S. Hershey Medical Center, Hershey, Pennsylvania; 7 Department of Family and Community Medicine, Penn State Health Milton S. Hershey Medical Center, Pennsylvania

## Abstract

**Importance:**

Infection has been postulated as a driver in the sudden infant death syndrome (SIDS) cascade. Epidemiologic patterns of infection, including respiratory syncytial virus and influenza, were altered during the COVID-19 pandemic. Comparing month-to-month variation in both sudden unexpected infant death (SUID) and SIDS rates before and during the pandemic offers an opportunity to generate and expand existing hypotheses regarding seasonal infections and SUID and SIDS.

**Objective:**

To compare prepandemic and intrapandemic rates of SUID and SIDS, assessing for monthly variation.

**Design, Setting, and Participants:**

This cross-sectional study assessed US mortality data provided by the Centers for Disease Control and Prevention for January 1, 2018, through December 31, 2021. Events with *International Statistical Classification of Diseases and Related Health Problems, Tenth Revision *codes for SIDS (R95), unknown (R99), and accidental suffocation and strangulation in bed (W75) causes of death were examined. The data analysis was performed between November 2, 2023, and June 2, 2024.

**Exposure:**

COVID-19 pandemic.

**Main Outcomes and Measures:**

The primary and secondary outcomes were the monthly rates of SUID and SIDS during the COVID-19 pandemic (March 1, 2020, to December 31, 2021) compared with the prepandemic period (March 1, 2018, to December 31, 2019) as measured using generalized linear mixed-effects models. Seasonal trends in RSV and influenza rates were also examined.

**Results:**

There were 14 308 SUID cases from January 1, 2018, to December 31, 2021 (42% female infants). Compared with the prepandemic period, the risk of SUID increased during the intrapandemic period (intensity ratio [IR], 1.06; 95% CI, 1.05-1.07). Monthly assessments revealed an increased risk of SUID beyond the prepandemic baseline starting in July 2020, with a pronounced epidemiologic shift from June to December 2021 (ranging from 10% to 14%). Rates of SIDS were elevated throughout the intrapandemic period compared with the prepandemic baseline, with the greatest increase in July 2021 (IR, 1.18; 95% CI, 1.13-1.22) and August 2021 (IR, 1.17; 95% CI, 1.13-1.22). Seasonal shifts in RSV hospitalizations correlated with monthly changes in SUID observed during 2021.

**Conclusions and Relevance:**

This cross-sectional study found increased rates of both SUID and SIDS during the COVID-19 pandemic, with a significant shift in epidemiology from the prepandemic period noted in June to December 2021. These findings support the hypothesis that off-season resurgences in endemic infectious pathogens may be associated with SUID rates, with RSV rates in the US closely approximating this shift. Further investigation into the role of infection in SUID and SIDS is needed.

## Key Points

**Question:**

What is the association between the COVID-19 pandemic and sudden unexpected infant death (SUID) and sudden infant death syndrome (SIDS)?

**Findings:**

In this cross-sectional study of 14 308 SUID cases, the risk of SUID and SIDS increased during the intrapandemic period (March 2020 to December 2021) compared with the prepandemic period (March 2018 to December 2019), with the greatest increases noted in 2021 (9% for SUID and 10% for SIDS). A marked statistically significant monthly increase in SUID from June to December 2021 was observed.

**Meaning:**

These findings suggest that the pronounced shift in SUID epidemiology during the second year of the COVID-19 pandemic may be associated with altered infectious disease transmission at the time.

## Teaser

This cross-sectional study uses US mortality data to assess monthly rates of sudden unexpected infant death and sudden infant death syndrome before and during the COVID-19 pandemic.

## Introduction

An estimated 3400 infants die unexpectedly in the US each year, making sudden unexpected infant death (SUID) a leading cause of death in infants.^1,2,3^ Sudden unexpected infant death is an umbrella term used to describe the sudden death of an infant younger than 1 year for whom the cause of death is not apparent prior to investigation. Deaths from sudden infant death syndrome (SIDS), which occur during sleep and remain unexplained after a thorough postmortem investigation,^4^ comprise more than one-third of SUID cases.^3^ The exact pathogenesis of SIDS remains unknown, with one hypothesis being a triple-risk model that includes a multifactorial process combining an infant’s vulnerability at a critical stage in development with 1 or more environmental triggers.^5^ Risk factors include young maternal age, maternal smoking, late or no prenatal care, prematurity or low infant birth weight, male sex, prone or side sleeping position, soft sleeping surface, and overheating.^5,6,7,8^ Infection has been postulated to play a role in the SIDS cascade, as antecedent infection has been described in many deaths^9,10,11^ and SIDS historically peaked in winter months in temperate regions when endemic respiratory viruses circulate.^12^

The COVID-19 pandemic has had large- and small-scale adverse outcomes for health care systems and family well-being. Infants and children, though they tend to have milder COVID-19 symptoms, are not immune to growing and developing in an atypical and ever-changing environment.^13,14,15^ While it is possible that COVID-19 itself may serve as an external trigger for SUID,^16,17,18^ the pandemic may also have exacerbated or reduced risk factors and protective factors for SUID, including changes in the temporal epidemiology of other infections. Thus, the context of the COVID-19 pandemic provides a unique opportunity to generate hypotheses regarding the role of infection in SUID and SIDS.

From 2019 to 2020, the incidence of SIDS increased in the US,^2^ although SUID rates remained largely stable, possibly due to changes in diagnostic coding, as suggested by Shapiro-Mendoza et al.^19^ In 2021, SIDS rates again increased, but more in-depth analyses are needed.^1^ Incidence of SUID and SIDS assessed by year overlooks important, more granular changes that occurred during the pandemic based on surges in the number of COVID-19 cases or unintended consequences of pandemic mitigation measures that may have affected other factors, such as the circulation of endemic infections. Our study examines rates of SUID, including SIDS, from 2018 to 2021, comparing monthly trends between the prepandemic and intrapandemic periods, to better assess the association of the COVID-19 pandemic with changes in SUID.

## Methods

### Study Design and Dataset

This cross-sectional study used US mortality data from all 50 states and the District of Columbia between January 1, 2018, and December 31, 2021, provided by the Centers for Disease Control and Prevention (CDC) National Vital Statistics System.^20^ Data were provided to The Pennsylvania State University through the CDC Data Use Agreement for Vital Statistics. The Pennsylvania State University Institutional Review Board considered the study protocol not human participants research and waived the need for informed consent as this research analyzed publicly available, deidentified data. The study follows the Strengthening the Reporting of Observational Studies in Epidemiology (STROBE) reporting guideline.^21^

The data came from death certificates that contained a single underlying cause of death and demographic data. Race and ethnicity coding was revised in 2021 and, thus, could not be analyzed for 2021. Sudden unexpected infant death included 3 cause-of-death classifications using *International Statistical Classification of Diseases and Related Health Problems, Tenth Revision* (*ICD-10*) codes R95 (SIDS), R99 (unknown cause), and W75 (accidental suffocation and strangulation in bed [ASSB]). In the US, infant deaths are investigated by the medicolegal death investigation system (which includes medical examiners and coroners), with wide variability among jurisdictions.^22^ Diagnostic shifting has been reported with temporal decreases in SIDS codes accompanied by increases in ASSB and unknown cause codes and may be due to classification changes by death scene investigators. Thus, in addition to analyzing cause-specific death rates, we focused on the overall SUID rate, which may be less likely to be influenced by coding changes. The natality data used to calculate rates per 100 000 live births were extracted from CDC Wide-Ranging Online Data for Epidemiologic Research.^23^

### Outcomes

The primary outcome was the monthly difference in the rate of SUID during the COVID-19 pandemic (intrapandemic) compared with the prepandemic period. The secondary outcome was monthly differences in the intrapandemic vs prepandemic rate of SIDS. The outcomes were reported as intensity ratios (IRs). Risk of SUID and SIDS was calculated as 100 × (IR − 1), where IR is the ratio of the intrapandemic to the prepandemic rate of SUID and SIDS. Intensity ratio values greater than 1 indicate higher risk, and values less than 1 indicate lower risk.

### Generation of COVID-19, Influenza, and Respiratory Syncytial Virus Curves

Hospital rates for COVID-19, respiratory syncytial virus (RSV), and influenza per 100 000 were extracted from COVID-NET,^24^ RSV-NET,^25^ and FluSurv-NET,^26^ respectively. Data on RSV and influenza from January 2018 to December 2021 and COVID-19 from March 2020 to December 2021 are summarized. Weekly data were converted into monthly data for influenza. Only observations with age = all, race = all, and sex = all were used.

### Statistical Analysis

We calculated the overall and cause-specific SUID rates (SIDS, unknown cause, and ASSB) per 100 000 live births by year. We summarized the monthly count per 100 000 live births by using adjusted monthly rates (ie, the number of days in each month divided by the number of days in that year times the annual birth rate). These monthly rates were reported both numerically and graphically using fourth-degree polynomial regression curves created to fit monthly points for each year.

We defined the prepandemic period as March 1, 2018, to December 31, 2019, and the intrapandemic period as March 1, 2020, to December 31, 2021. These periods were selected to approximate the start of the COVID-19 pandemic in the US, as it was declared an emergency on March 13, 2020. We fit the entire period (from January 1, 2018, to December 31, 2021) to improve numerical stability and achieve better accuracy. We then applied a generalized linear mixed-effects model in the form of Poisson regression for the overall US analysis using the following model features: (1) the logarithm of the state’s adjusted natality as an offset; (2) random-effects for the state; (3) 4 centered, state-level explanatory variables: median age, Black-White ratio, Hispanic-White ratio, and male-female ratio; (4) a first-order autoregressive process to account for the correlation across the time intervals; (5) embedded cubic polynomial spline functions with 12 segments; and (6) knot points for splines selected at 4-month intervals. With *t* set as the elapsed number of months since January 2018 (ie, *t*[0] = 1), such that *t* ∈ (0, 48), the knot points were designated as *t*(1) = 4, *t*(2) = 8, *t*(3) = 12, *t*(4) = 16, *t*(5) = 20, *t*(6) = 24, *t*(7) = 28, *t*(8) = 32, *t*(9) = 36, *t*(10) = 40, and *t*(11) = 44.

We constructed an overall intrapandemic vs prepandemic comparison and 22 monthly intrapandemic vs prepandemic comparisons based on model estimates of SUID and SIDS rates. The ASSB and unknown cause rates could not be analyzed due to the unbalanced distribution of reporting across states, which impeded model stability. We did not adjust for multiple comparisons. Race and ethnicity were assessed in the model because they are known confounders in mortality outcomes. The results are reported as IRs, with statistical significance defined as 95% CIs that exclude 1. The data analyses were performed between November 2, 2023, and June 2, 2024, using R, version 4.2.3 (R Core Team) and SAS, version 9.4 (SAS Institute Inc) software.

## Results

**Figure 1.**
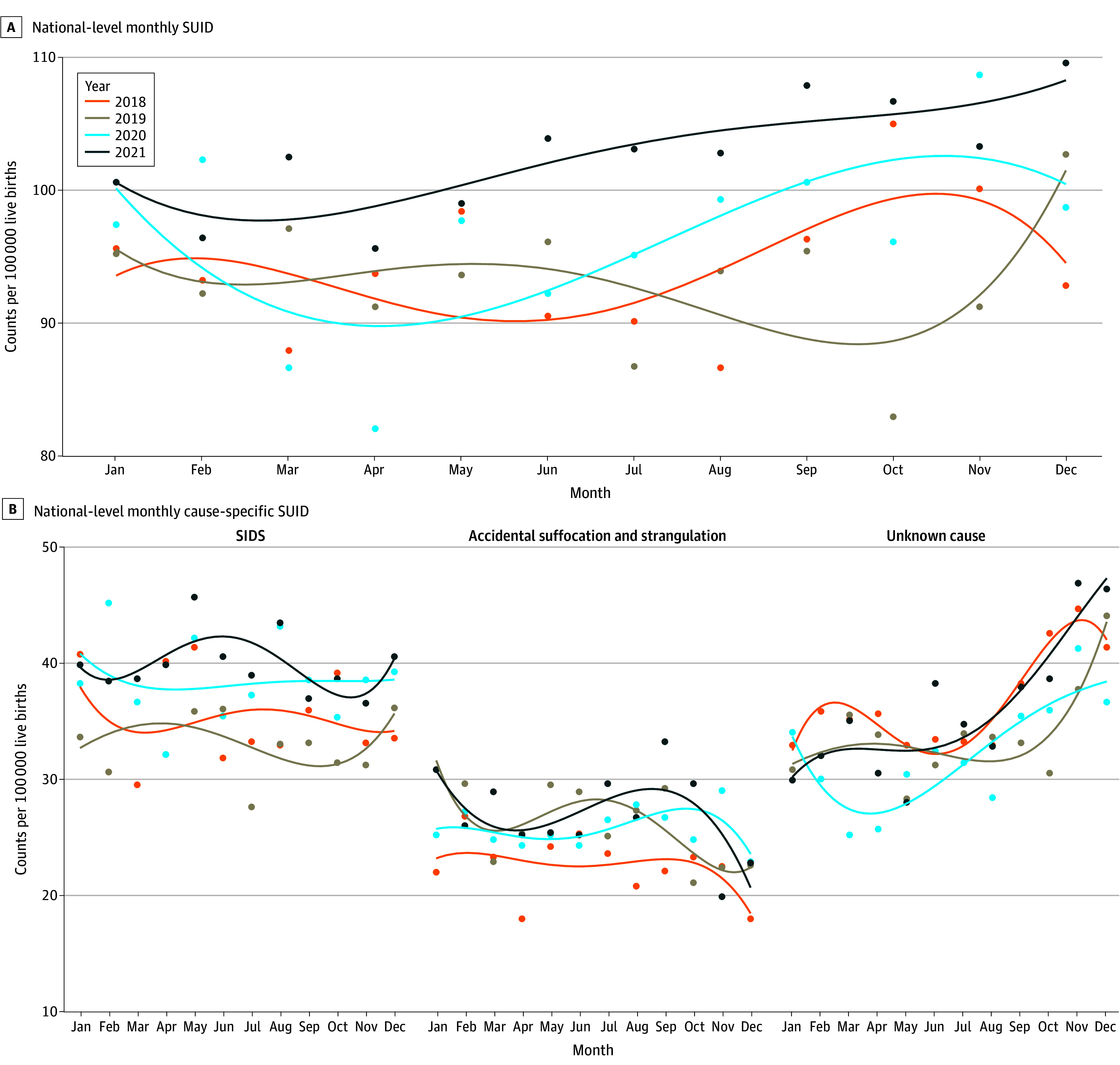
Monthly Sudden Unexpected Infant Death (SUID) and Cause-Specific SUID Rates by Year, 2018-2021 Cause-specific SUID included 3 underlying cause-of-death classifications coded per the *International Statistical Classification of Diseases and Related Health Problems, Tenth Revision*: sudden infant death syndrome (SIDS) (R95), unknown cause (R99), and accidental suffocation and strangulation in bed (W75). Monthly rates were calculated using adjusted monthly rates (ie, the number of days in each month divided by the number of days in that year times annual birth rate). Points indicate counts, and lines indicate the fourth-degree polynomial regression curves created to smooth the adjusted monthly rate for each year.

There were 14 308 cases of SUID from January 1, 2018, to December 31, 2021 (42% female and 58% male infants). Race and ethnicity data were available between 2018 and 2020 for 10 546 infants, with 2.5% reported as American Indian or Alaska Native, 2.4% as Asian, 38.6% as Black, and 56.5% as White (eTable 1 in Supplement 1). Cases of SUID per 100 000 live births increased from 94.2 in 2018 and 93.2 in 2019 to 96.4 in 2020 and 102.7 in 2021 (Table). Upon examination of specific causes of SUID, SIDS increased from 35.2 in 2018 and 33.3 in 2019 to 38.4 in 2020 and 39.8 in 2021. By monthly trends, the number of SUID and SIDS cases were generally higher in 2021 than in other years (Figure 1; eTables 2 and 3 in Supplement 1). Deaths coded as unknown cause and ASSB did not show a clear pattern (Table; Figure 1).

**Table.** Annual SUID and Cause-Specific SUID in the US, 2018-2021

Cause (*ICD-10* code)	No. of cases^a^				Cases per 100 000 live births^b^			
	2018	2019	2020	2021	2018	2019	2020	2021
Total SUID	3571	3493	3482	3762	94.2	93.2	96.4	102.7
SIDS (R95)	1334	1248	1389	1459	35.2	33.3	38.4	39.8
Unknown (R99)	1385	1263	1164	1315	36.5	33.7	32.2	35.9
ASSB (W75)	852	982	929	988	22.5	26.2	25.7	27.0

Abbreviations: ASSB, accidental suffocation and strangulation in bed; *ICD-10*, *International Statistical Classification of Diseases and Related Health Problems, Tenth Revision*; SIDS, sudden infant death syndrome; SUID, sudden unexpected infant death.

^a^ Cases for SUID and cause-specific SUID from US mortality data provided by the Centers for Disease Control and Prevention National Vital Statistics System.^20^

^b^ Live births were extracted from the Centers for Disease Control and Prevention Wide-Ranging Online Data for Epidemiologic Research.^23^

**Figure 2.**
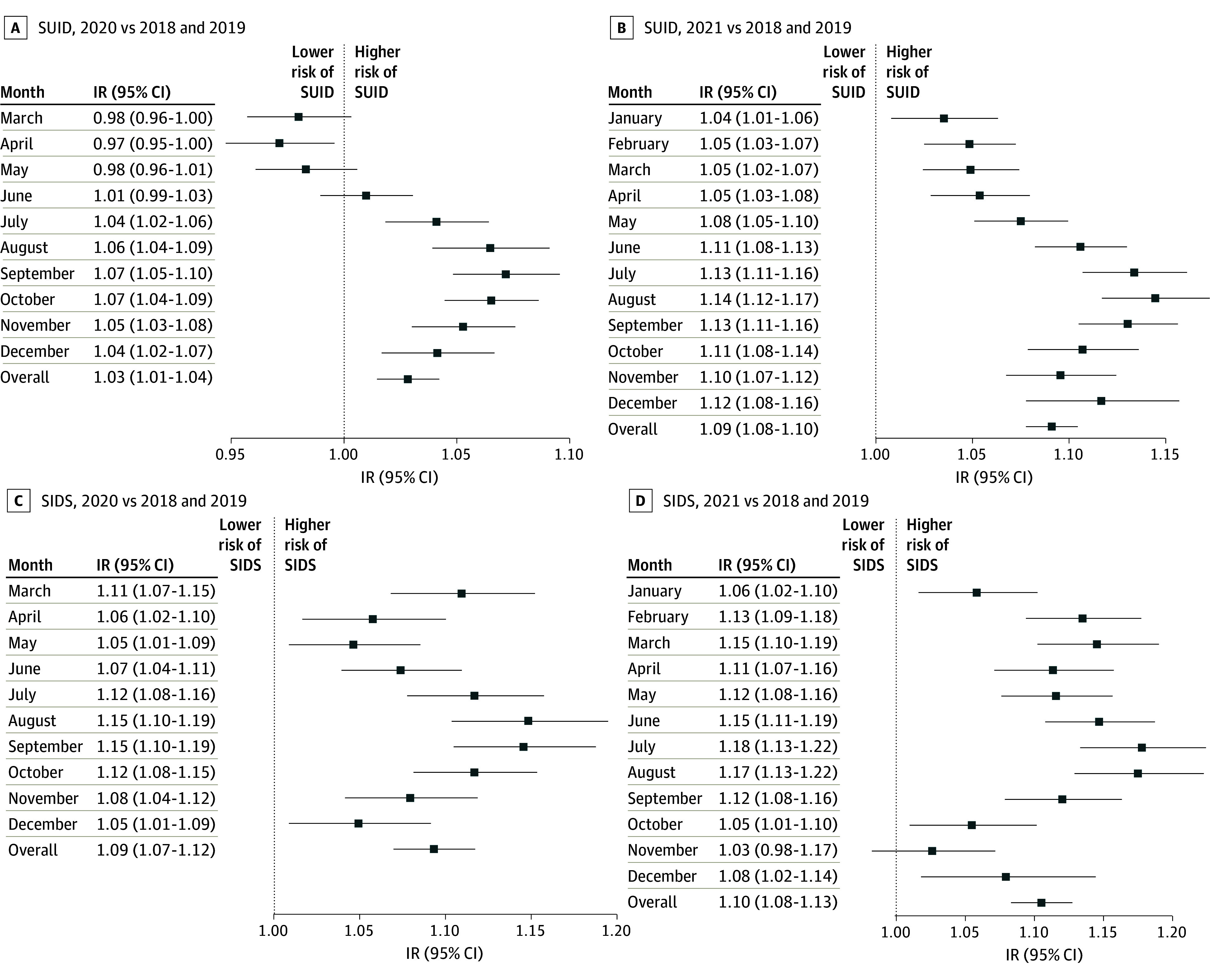
Monthly Risk of Sudden Unexpected Infant Death (SUID) and Sudden Infant Death Syndrome (SIDS) Between Intrapandemic and Prepandemic Periods The dashed line indicates the null estimate. An intensity ratio (IR) greater than 1.00 indicates higher rates of SUID or SIDS during the COVID-19 pandemic compared with the prepandemic period.

Comparisons of the pre- and intrapandemic periods using generalized linear mixed-effects models (eFigures 1 and 2 in Supplement 1) showed a higher risk of SUID during the intrapandemic period (IR, 1.06; 95% CI, 1.05-1.07). The risk of SIDS was also higher during the intrapandemic period (IR, 1.10; 95% CI, 1.08-1.12). The risk of SUID was higher in 2020 compared with the prepandemic period (IR, 1.03; 95% CI, 1.01-1.04) (Figure 2A; eTable 4 in Supplement 1). The risk of SUID cases was lower in April 2020 and then increased above the prepandemic baseline in July (IR, 1.04; 95% CI, 1.02-1.06), August (IR, 1.06; 95% CI, 1.04-1.09), September (IR, 1.07; 95% CI, 1.05-1.10), October (IR, 1.07; 95% CI, 1.04-1.09), November (IR, 1.05; 95% CI, 1.03-1.08), and December (IR, 1.04; 95% CI, 1.02-1.07). The risk of SIDS was also higher in 2020 compared with the prepandemic period (IR, 1.09; 95% CI, 1.07-1.12), increasing in every month from March to December 2020 (Figure 2C; eTable 5 in Supplement 1).

The risk of SUID was higher in 2021 compared with the prepandemic period (IR, 1.09; 95% CI, 1.08-1.10) (Figure 2B; eTable 6 in Supplement 1). While the SUID risk was high in every month, the most sizable increases were seen in June (IR, 1.11; 95% CI, 1.08-1.13), July (IR, 1.13; 95% CI, 1.11-1.16), August (IR, 1.14; 95% CI, 1.12-1.17), September (IR, 1.13; 95% CI, 1.11-1.16), October (IR, 1.11; 95% CI, 1.08-1.14), November (IR, 1.10; 95% CI, 1.07-1.12), and December (IR, 1.12; 95% CI, 1.08-1.16) of 2021. The risk of SIDS was also higher in 2021 (IR, 1.10; 95% CI, 1.08-1.13), and the number of cases each month was consistently elevated compared with the prepandemic period (except in November [IR, 1.03; 95% CI, 0.98-1.07]) (Figure 2D; eTable 7 in Supplement 1). Peak changes in SIDS were observed in July (IR, 1.18; 95% CI, 1.13-1.22) and August (IR, 1.17; 95% CI, 1.13-1.22) of 2021.

**Figure 3.**
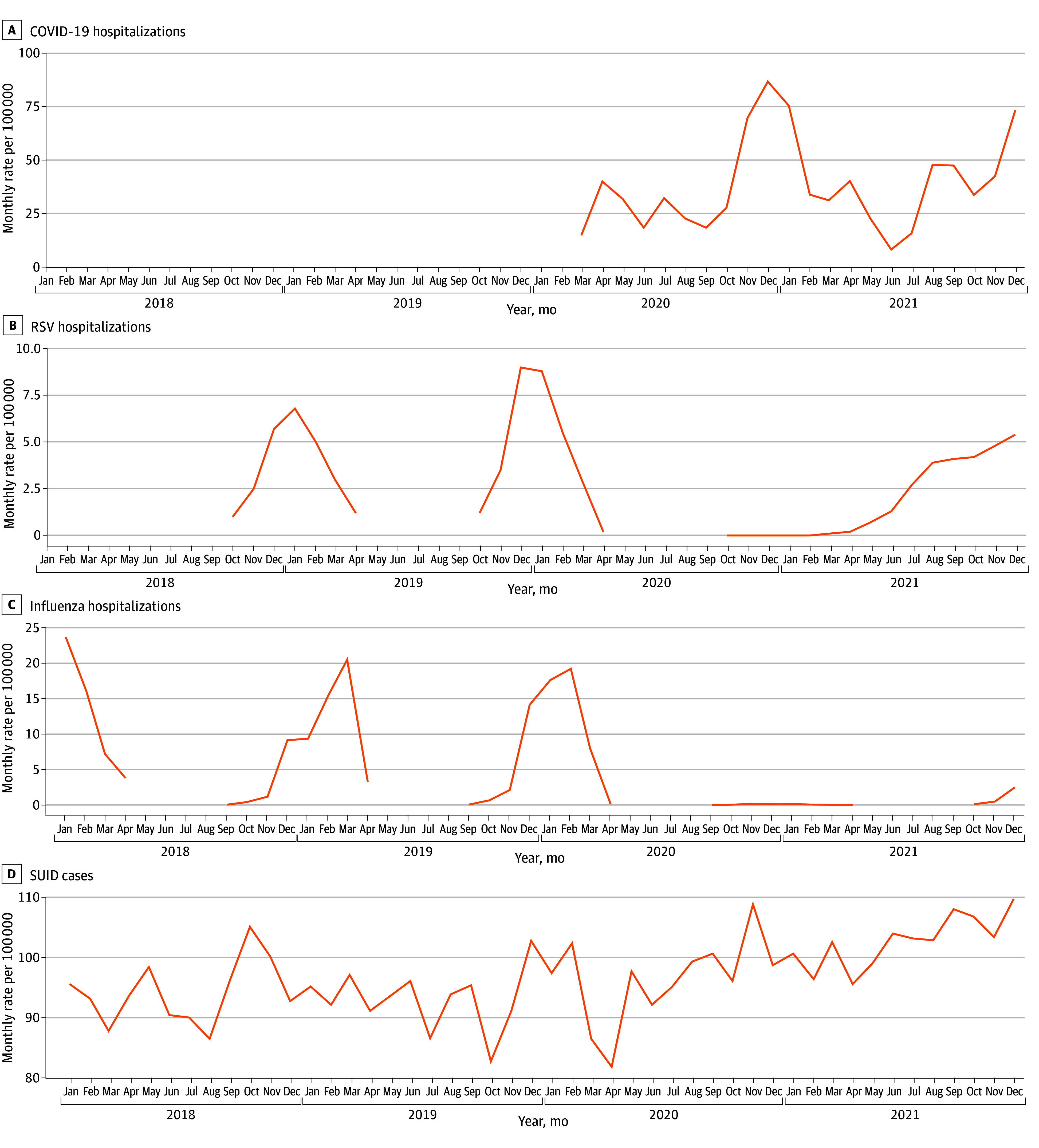
US Seasonal Respiratory Virus Hospitalization Rates and Sudden Unexpected Infant Death (SUID) Cases, 2018-2021 RSV indicates respiratory syncytial virus.

When generating hospitalization curves for influenza and RSV, prepandemic hospitalization rates followed a seasonal pattern in winter months (Figure 3). During the intrapandemic period, few RSV hospitalizations were described in 2020 but then were described off season in 2021 (June to December). Influenza hospitalizations were rarely described throughout both 2020 and 2021. COVID-19 hospitalizations had no clear association with monthly changes in SUID rates.

## Discussion

This cross-sectional study shows an increase in SUID and SIDS in the US during the COVID-19 intrapandemic vs prepandemic period. The increase was most pronounced during 2021 (rates of 9% for SUID and 10% for SIDS), and there was a profound monthly shift seen from June to December 2021 (ranging from 10% to 14% for SUID and 3% to 18% for SIDS). This change in the epidemiology of SUID in the context of the COVID-19 pandemic may advance current hypotheses or lay the groundwork for new theories regarding unexplained infant deaths.

Prior work on SUID by Shapiro-Mendoza et al^19^ identified an increase in SIDS rates in 2020 with relative consistency in SUID rates, suggestive of a diagnostic coding change. However, the authors found that non-Hispanic Black infants had consistently higher SUID rates during 2020, postulating that SUID disparities among non-Hispanic Black infants were exacerbated by the COVID-19 pandemic.^19^ While we were unable to assess race and ethnicity in our analysis, the monthly increase in SUID cases starting in July 2020 and continuing throughout 2021 may fit with the unintended cumulative outcomes of pandemic mitigation strategies that disrupted social networks, limited access to health care, changed childcare accommodations, and led to other social, economic, and emotional stressors. As the triple-risk model suggests, the pathogenesis of SIDS may involve a complicated interplay of factors.^5^ In this regard, we found a more substantial change in both SUID and SIDS rates starting in June 2021 as pandemic mitigation measures relaxed in the US. Accumulating social and economic consequences associated with the pandemic may have contributed, yet it is interesting that as social disruption lessened, unexplained infant deaths rose. One hypothesis is that a novel exogenous stressor, such as an infectious pathogen, may have played a role.

Pandemic mitigation measures interrupted transmission of endemic respiratory illnesses as evidenced by historic US lows of influenza and RSV cases in 2020.^27,28^ As COVID-19 vaccines became available in 2021, pandemic precautions were lifted across the US, with schools and workplaces reopening and social gatherings resuming.^29^ As a result, the second year of the pandemic saw resurgences of seasonal respiratory viruses at unexpected times and with increased severity.^27,30^ This resurgence was particularly extensive in infants and small children, who were increasingly susceptible due to a combination of waning maternal immunity and lack of individual exposure.^31,32^ Alongside this temporal change in endemic viruses, our analysis revealed a pronounced increase in SUID cases in the summer and autumn of 2021.

Mild upper respiratory viral infections have been reported in analyses of SIDS cases,^9,33,34^ and risk factors for SIDS, such as male sex, prematurity, low birth weight, inadequate prenatal care, higher birth order, cigarette smoke exposure, lack of breastfeeding, low socioeconomic status, and daycare attendance, have been found to parallel risks for infection.^7,35^ In addition, historically there existed a seasonality of SIDS in temperate regions, with winter increases that correlate with seasonal peaks of respiratory illnesses, although this has been less clear in recent analyses and may be confounded by heating of homes and bedding practices.^12,36,37,38^ One pathogen of interest in SIDS pathogenesis is RSV, as it infects nearly all children by age 2 years and is the leading cause of infant bronchiolitis, resulting in an estimated 80 000 annual US hospitalizations.^39^ Respiratory syncytial virus has been isolated in the lungs of infants with SIDS,^40,41^ though available diagnostic techniques and retrospective data have prevented any causal determination. When looking at RSV hospitalization trends in the US during the pandemic, there was a marked departure from the expected seasonality. Typically, RSV cases occur between October and April and peak in December and January. Few cases of RSV were reported in 2020, but RSV then resurged in June 2021 with a peak in August, making the 2021 RSV season 20 weeks earlier than usual.^39^ This shift in RSV hospitalizations correlated with monthly changes in SUID observed during 2021, while COVID-19 peaks and influenza hospitalizations did not.

If RSV plays a role in SUID, then SUID may be driven by the infection itself or by an interplay with other factors, such as environmental, genetic, or their combination. While RSV has a tropic effect at the respiratory tract, there is no clearly established link between temporal trends in bronchiolitis and the occurrence of SUID, suggesting that other factors may be at play.^42,43,44^ Potentially, RSV may mediate infant death via extrapulmonary manifestations, leading to cardiac arrhythmias or causing central apnea or delayed airway-protective reflexes.^45,46,47^ The association between SUID and RSV may also be influenced by bacterial colonization of the nasopharynx.^11,35^ The bacterial toxin model of SIDS pathogenesis posits that viral infection along with prone positioning may induce a favorable environment for increased bacterial toxin production that potentiates death in susceptible infants.^40,48,49,50^

Respiratory syncytial virus may also be a bridge within the triple-risk model, combining the mechanistic theories discussed above with aberrant inflammatory responses in an infant with genetic susceptibility. Increased markers of inflammation have been found in SIDS autopsy specimens, and increased immunoglobulins and T-cell activation markers at mucosal surfaces have been described.^40,51^ In addition, a host of gene variants have been associated with SIDS that are presumed to confer defects in immune regulation via interleukin and cytokine signaling, along with activation of the complement cascade.^50,51,52^ One noteworthy cytokine found in the cerebrospinal fluid in SIDS cases is IL-6, which induces fevers and may lead to alterations in infant respiration, mediating apneic episodes.^53,54^ Altogether, an aberrant immune cascade triggered by RSV in combination with other external factors, such as prone positioning or maternal smoking, may play a central role in SUID.

### Limitations

Our analysis has several important limitations. First, our study uses CDC mortality rather than linked infant birth and death data, limiting our ability to assess the number of infants at risk in any given month. This limitation may also account for small differences between our dataset and SUID rates previously reported in a similar period.^19^ In addition, available data were limited by state reporting, resulting in model instability for ASSB and unexplained deaths. Second, race and ethnicity reporting was variable and incomplete; thus, we could not ascertain whether epidemiologic shifts were seen disproportionately in racial and ethnic minority groups known to experience disparate SUID rates.^55^ These challenges are compounded by the wide range of unexpected infant death investigation and reporting practices in the US, which may influence the robustness of analyses and make it challenging to draw meaningful conclusions.^38^ Third, when considering the association of an unmasked infectious contributor to SUID, we were unable to decipher correlation vs causation due to the retrospective nature of available data, inability to assess confounders, and lack of consistent pediatrician input into the death evaluation, which may lead to over- or underidentification of the role of infection. Fourth, in terms of confounders, we were unable to assess other risk factors for SIDS, including smoking, breastfeeding,^56^ changes in childcare use,^57^ health care access,^58^ and others, that were almost all certainly impacted by the COVID-19 pandemic. Fifth, if infection was the sole risk factor for SIDS, we did not observe an anticipated drop in rates in 2020 when respiratory viruses, such as RSV, were not circulating. Finally, RSV is just one of many pathogens with a change in seasonality during the pandemic. Observed trends of influenza in this study did not correlate with the shift in SUID rates, but other infections that lack consistent testing and reporting in the US may have contributed.^27^

## Conclusions

In this cross-sectional study of SUID cases from 2018 to 2021, we noted an increase in 2021 SUID and SIDS rates during the COVID-19 pandemic, with a significant epidemiologic shift from the prepandemic period. While other studies have shown changes in infant death rates during the pandemic,^59,60,61^ none to our knowledge have examined these changes by month or attempted to overlay risk factors that may be associated with the pandemic, such as respiratory virus epidemiology. Our observational data show an epidemiologic shift of SUID and SIDS during the pandemic where a plausible unifying hypothesis may be an infectious stimulus contributing to a proportion of cases. While our assessment is merely hypothetical and meant to be hypothesis generating, if a clear infectious contributor to SIDS were identified, mitigation efforts might incorporate infection prevention strategies to protect vulnerable infants beyond the currently used safe sleep practices. Future work in the short term might investigate unexpected infant death rates in other countries in conjunction with respiratory virus epidemiology. In the longer term, dedicated observational studies or the incorporation of SUID rates into existing respiratory virus or pandemic preparedness networks might allow further investigation of associations between infection and SUID. It may be particularly interesting to evaluate SUID rates following the rollout of several new RSV countermeasures, including maternal RSV vaccination and infant nirsevimab administration. Data such as ours may help theorize plausible causal pathways and potential mitigable risk factors to further reduce the risk of SUID.

## Notes

Supplement 1. **eTable 1.** Summary Demographic Information of 14 308 Sudden Unexpected Infant Death (SUID) Cases, 2018-2021 **eTable 2.** Distribution of Sudden Unexpected Infant Death (SUID) Cases and Adjusted Rate per 100 000 Live Births by Month, 2018-2021 **eTable 3.** Distribution of Sudden Infant Death Syndrome (SIDS) Cases and Adjusted Rate per 100 000 Live Births by Month, 2018-2021 **eFigure 1.** Monthly Sudden Unexpected Infant Death (SUID) From January 2018 to December 2021 **eFigure 2.** Monthly Sudden Infant Death Syndrome (SIDS) From January 2018 to December 2021 **eTable 4.** Intensity Ratios (IRs) of Sudden Unexpected Infant Death (SUID) in March 2020 to December 2020 Compared With 2018 and 2019 **eTable 5.** Intensity Ratios (IRs) of Sudden Infant Death Syndrome (SIDS) in March 2020 to December 2020 Compared With 2018 and 2019 **eTable 6.** Intensity Ratios (IRs) of Sudden Unexpected Infant Death (SUID) in January 2021 to December 2021 Compared With 2018 and 2019 **eTable 7.** Intensity Ratios (IRs) of Sudden Infant Death Syndrome (SIDS) in January 2021 to December 2021 Compared With 2018 and 2019

Supplement 2.
Data Sharing Statement
