# Supplementary material for: Rates of Sudden Unexpected Infant Death Before and During the COVID-19 Pandemic
Source: JAMA Netw Open. 2024 Sep 26;7(9):e2435722. doi: 10.1001/jamanetworkopen.2024.35722 (PMC11427960; doi:10.1001/jamanetworkopen.2024.35722)
Supplement: Supplement 1. — eTable 1. Summary Demographic Information of 14 308 Sudden Unexpected Infant Death (SUID) Cases, 2018-2021 eTable 2. Distribution of Sudden Unexpected Infant Death (SUID) Cases and Adjusted Rate per 100 000 Live Births by Month, 2018-2021 eTable 3. Distribution of Sudden Infant Death Syndrome (SIDS) Cases and Adjusted Rate per 100 000 Live Births by Month, 2018-2021 eFigure 1. Monthly Sudden Unexpected Infant Death (SUID) From January 2018 to December 2021 eFigure 2. Monthly Sudden Infant Death Syndrome (SIDS) From January 2018 to December 2021 eTable 4. Intensity Ratios (IRs) of Sudden Unexpected Infant Death (SUID) in March 2020 to December 2020 Compared With 2018 and 2019 eTable 5. Intensity Ratios (IRs) of Sudden Infant Death Syndrome (SIDS) in March 2020 to December 2020 Compared With 2018 and 2019 eTable 6. Intensity Ratios (IRs) of Sudden Unexpected Infant Death (SUID) in January 2021 to December 2021 Compared With 2018 and 2019 eTable 7. Intensity Ratios (IRs) of Sudden Infant Death Syndrome (SIDS) in January 2021 to December 2021 Compared With 2018 and 2019 [file jamanetwopen-e2435722-s001.pdf]

Supplemental Online Content

Guare EG, Zhao R, Ssentongo P, Batra EK, Chinchilli VM, Paules CI. Rates of sudden unexpected infant death before and during the COVID-19 pandemic. *JAMA Netw Open*. 2024;7(9):e2435722. doi:10.1001/jamanetworkopen.2024.35722

eTable 1. Summary Demographic Information of 14 308 Sudden Unexpected Infant Death (SUID) Cases, 2018-2021

eTable 2. Distribution of Sudden Unexpected Infant Death (SUID) Cases and Adjusted Rate per 100 000 Live Births by Month, 2018-2021

eTable 3. Distribution of Sudden Infant Death Syndrome (SIDS) Cases and Adjusted Rate per 100 000 Live Births by Month, 2018-2021

eFigure 1. Monthly Sudden Unexpected Infant Death (SUID) From January 2018 to December 2021

eFigure 2. Monthly Sudden Infant Death Syndrome (SIDS) From January 2018 to December 2021

eTable 4. Intensity Ratios (IRs) of Sudden Unexpected Infant Death (SUID) in March 2020 to December 2020 Compared With 2018 and 2019

eTable 5. Intensity Ratios (IRs) of Sudden Infant Death Syndrome (SIDS) in March 2020 to December 2020 Compared With 2018 and 2019

eTable 6. Intensity Ratios (IRs) of Sudden Unexpected Infant Death (SUID) in January 2021 to December 2021 Compared With 2018 and 2019

eTable 7. Intensity Ratios (IRs) of Sudden Infant Death Syndrome (SIDS) in January 2021 to December 2021 Compared With 2018 and 2019

This supplemental material has been provided by the authors to give readers additional information about their work.

© 2024 Guare EG et al. *JAMA Network Open*.

eTable 1. Summary Demographic Information of 14 308 Sudden Unexpected Infant Death (SUID) Cases, 2018-2021

	2018	2019	2020	2021[a]	Total
**Total SUID**	3571	3493	3482	3762	14308
**Sex, n (%)**					
Female	1471 (41)	1462 (42)	1494 (43)	1624 (43)	6051 (42)
Male	2100 (59)	2031 (58)	1988 (57)	2138 (57)	8257 (58)
**Race/ethnicity, n (%)**					
White	2076 (58)	1970 (56)	1914 (55)	NA	
Black	1305 (37)	1339 (38)	1424 (41)	NA	
American Indian/Alaska Native	91 (2.5)	98 (2.8)	79 (2.3)	NA	
Asian	99 (2.8)	86 (2.5)	65 (1.9)	NA	

a: Race/ethnicity data missing for 2021.
Abbreviations: NA not applicable

eTable 2. Distribution of Sudden Unexpected Infant Death (SUID) Cases and Adjusted Rate per 100 000 Live Births by Month, 2018-2021

	2018	2019	2020	2021
**January**	308 (95.6)	303 (95.2)	298 (97.4)	313 (100.6)
**February**	271 (93.2)	265 (92.2)	293 (102.3)	271 (96.4)
**March**	283 (87.9)	309 (97.1)	265 (86.6)	319 (102.5)
**April**	292 (93.7)	281 (91.2)	243 (82.0)	288 (95.6)
**May**	317 (98.4)	398 (93.6)	299 (97.7)	308 (99.0)
**June**	282 (90.5)	296 (96.1)	273 (92.2)	313 (103.9)
**July**	290 (90.1)	276 (86.7)	291 (95.1)	321 (103.1)
**August**	279 (86.6)	299 (93.9)	304 (99.3)	320 (102.8)
**September**	300 (96.3)	294 (95.4)	298 (100.6)	325 (107.9)
**October**	338 (105.0)	264 (82.9)	294 (96.1)	332 (106.7)
**November**	312 (100.1)	281 (91.2)	322 (108.7)	311 (103.3)
**December**	299 (92.8)	327 (102.7)	302 (98.7)	341 (109.6)

Cases (adjusted rate per 100,000)

© 2024 Guare EG et al. *JAMA Network Open*.

eTable 3. Distribution of Sudden Infant Death Syndrome (SIDS) Cases and Adjusted Rate per 100 000 Live Births by Month, 2018-2021

	2018	2019	2020	2021
**January**	131 (40.7)	107 (33.6)	117 (38.2)	124 (39.8)
**February**	89 (30.6)	88 (30.6)	129 (45.1)	108 (38.4)
**March**	95 (29.5)	123 (38.6)	112 (36.6)	120 (38.6)
**April**	125 (40.1)	99 (32.1)	95 (32.1)	120 (39.8)
**May**	133 (41.3)	114 (35.8)	129 (42.1)	142 (45.6)
**June**	99 (31.8)	111 (36.0)	105 (35.4)	122 (40.5)
**July**	107 (33.2)	88 (27.6)	114 (37.2)	121 (38.9)
**August**	106 (32.9)	105 (33.0)	132 (43.1)	135 (43.4)
**September**	112 (35.9)	102 (33.1)	114 (38.5)	111 (36.9)
**October**	126 (39.1)	100 (31.4)	108 (35.3)	120 (38.6)
**November**	103 (33.1)	96 (31.2)	114 (38.5)	111 (36.5)
**December**	108 (33.5)	115 (36.1)	120 (39.4)	126 (40.5)

Cases (adjusted rate per 100,000)

© 2024 Guare EG et al. *JAMA Network Open*.

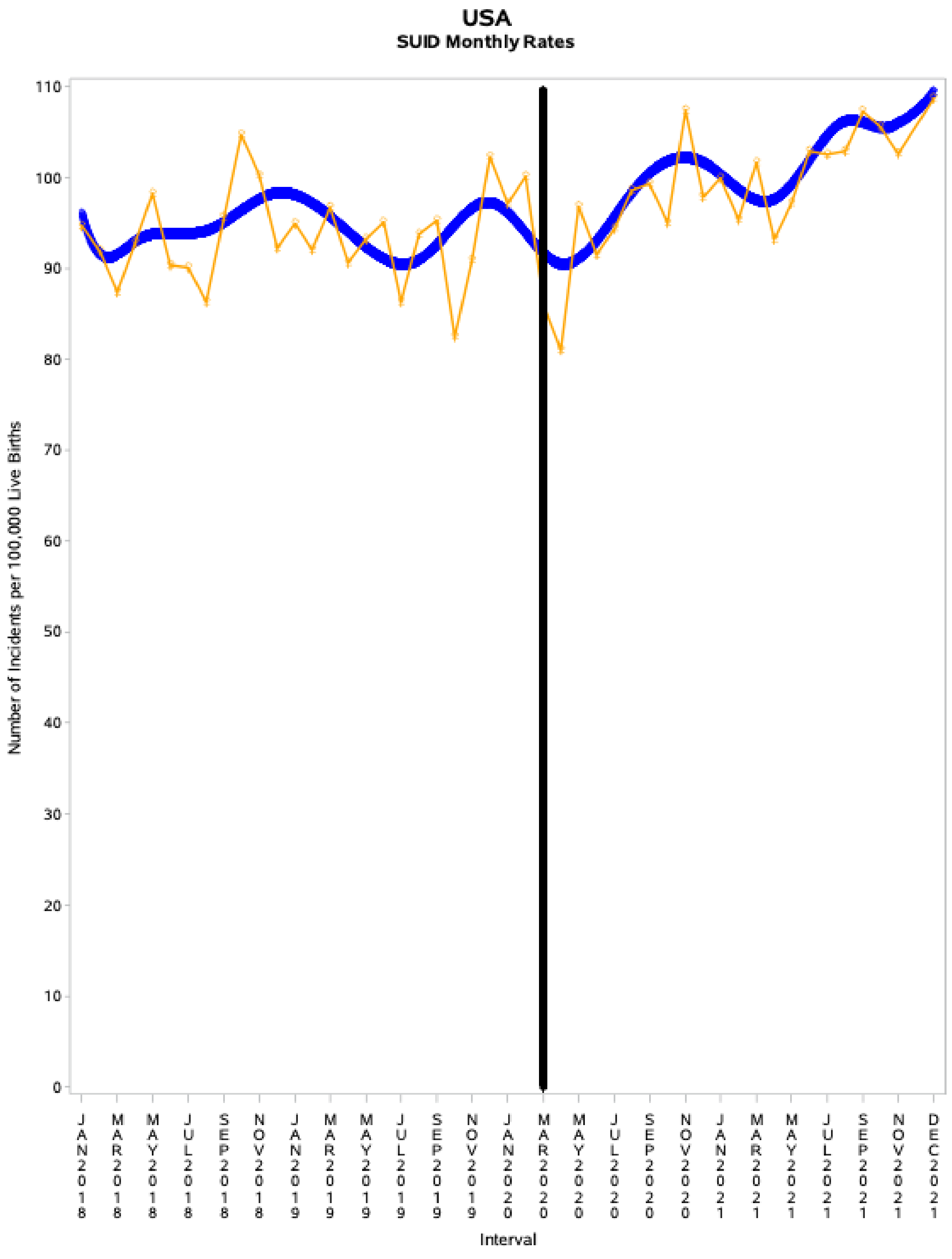

eFigure 1. Monthly Sudden Unexpected Infant Death (SUID) From January 2018 to December 2021. The orange line represents monthly SUID cases per 100,000 live births. The blue line represents model-based estimated monthly SUID cases per 100,000 live births. The vertical black line shows March 2020, or the beginning of the pandemic period.

© 2024 Guare EG et al. *JAMA Network Open*.

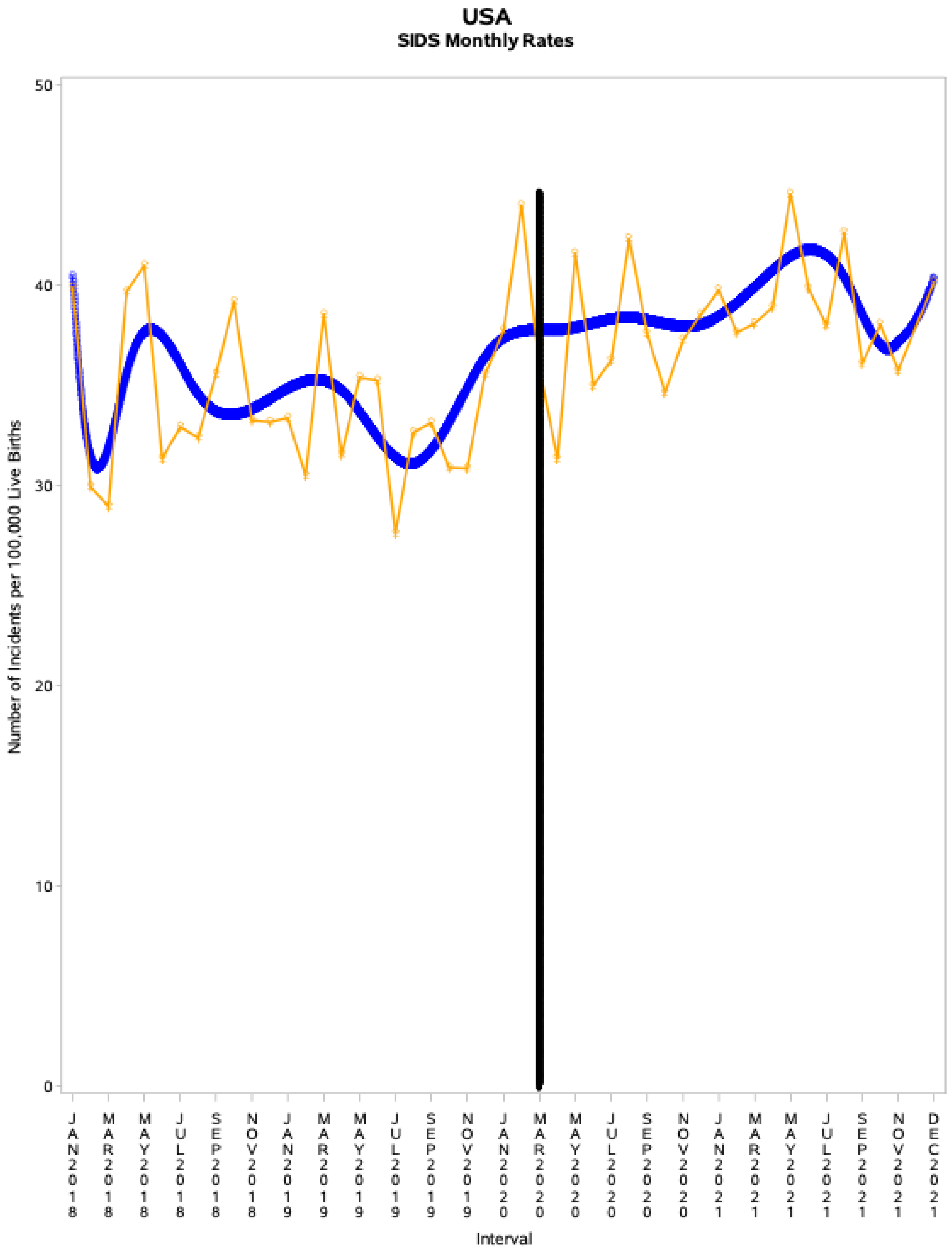

eFigure 2. Monthly Sudden Infant Death Syndrome (SIDS) From January 2018 to December 2021. The orange line represents monthly SIDS cases per 100,000 live births. The blue line represents model-based estimated monthly SIDS cases per 100,000 live births. The vertical black line shows March 2020, or the beginning of the pandemic period.

© 2024 Guare EG et al. *JAMA Network Open*.

eTable 4. Intensity Ratios (IRs) of Sudden Unexpected Infant Death (SUID) in March 2020 to December 2020 Compared With 2018 and 2019

Month	Intensity Ratio	Standard Deviation	Lower Bound	Upper Bound	P-value
**March**	0.98	0.01	0.96	1.00	0.96
**April**	0.97	0.01	0.95	1.00	0.99
**May**	0.98	0.01	0.96	1.01	0.93
**June**	1.01	0.01	0.99	1.03	0.18
**July**	1.04	0.01	1.02	1.06	<0.001
**August**	1.06	0.01	1.04	1.09	<0.001
**September**	1.07	0.01	1.05	1.10	<0.001
**October**	1.07	0.01	1.04	1.09	<0.001
**November**	1.05	0.01	1.03	1.08	<0.001
**December**	1.04	0.01	1.02	1.07	0.001
**Overall**	1.03	0.01	1.01	1.04	<0.001

eTable 5. Intensity Ratios (IRs) of Sudden Infant Death Syndrome (SIDS) in March 2020 to December 2020 Compared With 2018 and 2019

Month	Intensity Ratio	Standard Deviation	Lower Bound	Upper Bound	P-value
**March**	1.11	0.02	1.07	1.15	<0.001
**April**	1.06	0.02	1.02	1.10	<0.001
**May**	1.05	0.02	1.01	1.09	0.003
**June**	1.07	0.02	1.04	1.11	<0.001
**July**	1.12	0.02	1.08	1.16	<0.001
**August**	1.15	0.02	1.10	1.19	<0.001
**September**	1.15	0.02	1.10	1.19	<0.001
**October**	1.12	0.02	1.08	1.15	<0.001
**November**	1.08	0.02	1.04	1.12	<0.001
**December**	1.05	0.02	1.01	1.09	0.003
**Overall**	1.09	0.01	1.07	1.12	<0.001

© 2024 Guare EG et al. *JAMA Network Open*.

eTable 6. Intensity Ratios (IRs) of Sudden Unexpected Infant Death (SUID) in January 2021 to December 2021 Compared With 2018 and 2019

Month	Intensity Ratio	Standard Deviation	Lower Bound	Upper Bound	P-value
**January**	1.04	0.01	1.01	1.06	0.005
**February**	1.05	0.01	1.03	1.07	<0.001
**March**	1.05	0.01	1.02	1.07	<0.001
**April**	1.05	0.01	1.03	1.08	<0.001
**May**	1.08	0.01	1.05	1.10	<0.001
**June**	1.11	0.01	1.08	1.13	<0.001
**July**	1.13	0.01	1.11	1.16	<0.001
**August**	1.14	0.01	1.12	1.17	<0.001
**September**	1.13	0.01	1.11	1.16	<0.001
**October**	1.11	0.01	1.08	1.14	<0.001
**November**	1.10	0.01	1.07	1.12	<0.001
**December**	1.12	0.02	1.08	1.16	<0.001
**Overall**	1.09	0.01	1.08	1.10	<0.001

eTable 7. Intensity Ratios (IRs) of Sudden Infant Death Syndrome (SIDS) in January 2021 to December 2021 Compared With 2018 and 2019

Month	Intensity Ratio	Standard Deviation	Lower Bound	Upper Bound	P-value
**January**	1.06	0.02	1.02	1.10	<0.001
**February**	1.13	0.02	1.09	1.18	<0.001
**March**	1.15	0.02	1.10	1.19	<0.001
**April**	1.11	0.02	1.07	1.16	<0.001
**May**	1.12	0.02	1.08	1.16	<0.001
**June**	1.15	0.02	1.11	1.19	<0.001
**July**	1.18	0.02	1.13	1.22	<0.001
**August**	1.17	0.02	1.13	1.22	<0.001
**September**	1.12	0.02	1.08	1.16	<0.001
**October**	1.05	0.02	1.01	1.10	0.002
**November**	1.03	0.02	0.98	1.07	0.12
**December**	1.08	0.03	1.02	1.14	<0.001
**Overall**	1.10	0.01	1.08	1.13	<0.001

© 2024 Guare EG et al. *JAMA Network Open*.
