# Supplementary material for: Rates of Sudden Unexpected Infant Death Before and During the COVID-19 Pandemic
Source: JAMA Netw Open. 2024 Sep 26;7(9):e2435722. doi: 10.1001/jamanetworkopen.2024.35722 (PMC11427960; doi:10.1001/jamanetworkopen.2024.35722)
Supplement: Supplement 2. — Data Sharing Statement [file jamanetwopen-e2435722-s002.pdf]

Data Sharing Statement

Guare. Rates of Sudden Unexpected Infant Death Before and During the COVID-19 Pandemic. *JAMA Netw Open*. Published September 26, 2024. doi:10.1001/jamanetworkopen.2024.35722

Data
Data available: No

Additional Information
Explanation for why data not available: This data is available through public United States mortality data and can be requested through the Centers for Disease Control and Prevention.
